# Illness duration and symptom profile in symptomatic UK school-aged children tested for SARS-CoV-2

**DOI:** 10.1016/S2352-4642(21)00198-X

**Published:** 2021-10

**Authors:** Erika Molteni, Carole H Sudre, Liane S Canas, Sunil S Bhopal, Robert C Hughes, Michela Antonelli, Benjamin Murray, Kerstin Kläser, Eric Kerfoot, Liyuan Chen, Jie Deng, Christina Hu, Somesh Selvachandran, Kenneth Read, Joan Capdevila Pujol, Alexander Hammers, Tim D Spector, Sebastien Ourselin, Claire J Steves, Marc Modat, Michael Absoud, Emma L Duncan

**Affiliations:** a School of Biomedical Engineering and Imaging Sciences, Faculty of Life Sciences and Medicine, School of Life Course Sciences, King's College London, London, UK; b Department of Twin Research and Genetic Epidemiology, Faculty of Life Sciences and Medicine, School of Life Course Sciences, King's College London, London, UK; c Department of Women and Children's Health, Faculty of Life Sciences and Medicine, School of Life Course Sciences, King's College London, London, UK; d Department of Endocrinology, Guys and St Thomas' NHS Foundation Trust, London, UK; e Department of Ageing and Health, Guys and St Thomas' NHS Foundation Trust, London, UK; f MRC Unit for Lifelong Health and Ageing, Department of Population Health Sciences and Centre for Medical Image Computing, Department of Computer Science, University College London, London, UK; g Population Health Sciences Institute, Faculty of Medical Sciences, Newcastle University, Newcastle upon Tyne, UK; h Department of Population Health, Faculty of Epidemiology and Population Health, London School of Hygiene & Tropical Medicine, London, UK; i Zoe Limited, London, UK; j King's College London and Guy's and St Thomas' PET Centre, London, UK; k Children's Neurosciences, Evelina London Children' Hospital, St Thomas' Hospital, King's Health Partners, Academic Health Science Centre, London, UK

## Abstract

**Background:**

In children, SARS-CoV-2 infection is usually asymptomatic or causes a mild illness of short duration. Persistent illness has been reported; however, its prevalence and characteristics are unclear. We aimed to determine illness duration and characteristics in symptomatic UK school-aged children tested for SARS-CoV-2 using data from the COVID Symptom Study, one of the largest UK citizen participatory epidemiological studies to date.

**Methods:**

In this prospective cohort study, data from UK school-aged children (age 5–17 years) were reported by an adult proxy. Participants were voluntary, and used a mobile application (app) launched jointly by Zoe Limited and King's College London. Illness duration and symptom prevalence, duration, and burden were analysed for children testing positive for SARS-CoV-2 for whom illness duration could be determined, and were assessed overall and for younger (age 5–11 years) and older (age 12–17 years) groups. Children with longer than 1 week between symptomatic reports on the app were excluded from analysis. Data from symptomatic children testing negative for SARS-CoV-2, matched 1:1 for age, gender, and week of testing, were also assessed.

**Findings:**

258 790 children aged 5–17 years were reported by an adult proxy between March 24, 2020, and Feb 22, 2021, of whom 75 529 had valid test results for SARS-CoV-2. 1734 children (588 younger and 1146 older children) had a positive SARS-CoV-2 test result and calculable illness duration within the study timeframe (illness onset between Sept 1, 2020, and Jan 24, 2021). The most common symptoms were headache (1079 [62·2%] of 1734 children), and fatigue (954 [55·0%] of 1734 children). Median illness duration was 6 days (IQR 3–11) versus 3 days (2–7) in children testing negative, and was positively associated with age (Spearman's rank-order *r*_s_ 0·19, p<0·0001). Median illness duration was longer for older children (7 days, IQR 3–12) than younger children (5 days, 2–9). 77 (4·4%) of 1734 children had illness duration of at least 28 days, more commonly in older than younger children (59 [5·1%] of 1146 older children *vs* 18 [3·1%] of 588 younger children; p=0·046). The commonest symptoms experienced by these children during the first 4 weeks of illness were fatigue (65 [84·4%] of 77), headache (60 [77·9%] of 77), and anosmia (60 [77·9%] of 77); however, after day 28 the symptom burden was low (median 2 symptoms, IQR 1–4) compared with the first week of illness (median 6 symptoms, 4–8). Only 25 (1·8%) of 1379 children experienced symptoms for at least 56 days. Few children (15 children, 0·9%) in the negatively tested cohort had symptoms for at least 28 days; however, these children experienced greater symptom burden throughout their illness (9 symptoms, IQR 7·7–11·0 *vs* 8, 6–9) and after day 28 (5 symptoms, IQR 1·5–6·5 *vs* 2, 1–4) than did children who tested positive for SARS-CoV-2.

**Interpretation:**

Although COVID-19 in children is usually of short duration with low symptom burden, some children with COVID-19 experience prolonged illness duration. Reassuringly, symptom burden in these children did not increase with time, and most recovered by day 56. Some children who tested negative for SARS-CoV-2 also had persistent and burdensome illness. A holistic approach for all children with persistent illness during the pandemic is appropriate.

**Funding:**

Zoe Limited, UK Government Department of Health and Social Care, Wellcome Trust, UK Engineering and Physical Sciences Research Council, UK Research and Innovation London Medical Imaging and Artificial Intelligence Centre for Value Based Healthcare, UK National Institute for Health Research, UK Medical Research Council, British Heart Foundation, and Alzheimer's Society.

## Introduction

SARS-CoV-2 has caused more than 190 million cases of COVID-19 and 4 million deaths globally, as of July 21, 2021. In adults, SARS-CoV-2 causes a predominantly respiratory illness;^1^, ^2^ children are often asymptomatic (in 43–68% of cases) or have mild symptoms,^3^ and life-threatening illness and death from COVID-19 are rare.^4^, ^5^ The pandemic has also resulted in the emergence of a new rare condition, termed multisystem inflammatory syndrome in children (MIS-C),^6^ presenting at about 2–4 weeks after acute SARS-CoV-2 infection.^7^

**Research in context**

**Evidence before this study**

COVID-19 in children is usually asymptomatic or manifests as a mild illness of short duration. Concerns have been raised regarding prolonged illness in children, with no clear resolution of symptoms several weeks after onset, as is observed in some adults. How common this might be in children, the clinical features of such prolonged illness in children, and how this might compare with illnesses from other respiratory viruses (and with general population prevalence of these symptoms) is unclear. A final database search was done in PubMed using the search terms ((“COVID-19” OR “SARS-CoV-2”) AND child*) on June 28, 2021.

**Added value of this study**

We provide a detailed description of the illness duration and symptom burden for COVID-19 in UK school-aged children (age 5–17 years). Our data, collected in a digital surveillance platform through one of the largest UK citizen science epidemiological studies, show that long illness duration after SARS-CoV-2 infection in school-aged children does occur, but is uncommon. Only a small proportion of children had illness duration beyond 4 weeks, and their symptom burden decreased over time. Almost all children had symptom resolution by 8 weeks, providing reassurance about long-term outcomes. Additionally, the symptom burden in children with what has been termed long COVID-19 was not greater than that in children with long illnesses due to causes other than SARS-CoV-2 infection.

**Implications of all the available evidence**

Our data confirm that COVID-19 in UK school-aged children is usually of short duration and low symptom burden. Some children do have longer illness duration, and our findings validate their experiences; however, most of these children usually recover with time. Our findings emphasise that appropriate resources will be necessary for any child with prolonged illness, whether due to COVID-19 or other illnesses. Our study provides crucial data to inform discussions about the effect and implications of the pandemic on health-care resource allocation.

Some adults with COVID-19 experience prolonged illness duration, known as “long COVID”.^8^, ^9^ Longitudinal data from the King's College London COVID Symptom Study^10^ showed that 13·3% of adults with a positive test for SARS-CoV-2 had symptoms for at least 4 weeks (referred to as LC28) and 4·5% had symptoms for at least 8 weeks (LC56).^2^ Whether some children also experience prolonged illness duration,^9^, ^11^ and if so, how this compares with other illnesses, is unknown.

In September, 2020, coinciding with UK schools re-opening, governance for COVID Symptom Study data usage was extended to allow analysis of data from children. The UK subsequently experienced further pandemic waves, during which testing was widely available for individuals experiencing fever, cough, anosmia, or a combination of these symptoms, in contrast to the first wave when testing was mostly restricted to individuals presenting to hospital.^12^, ^13^ Stay-at-home directives and school closures over winter resulted in unusually low circulation of viruses such as influenza and adenovirus in the UK;^14^ however, individuals who contracted other respiratory illnesses were often tested for SARS-CoV-2 due to symptom overlap.

We aimed to investigate illness duration and symptom prevalence, duration, and burden in UK school-aged children (age 5–17 years) testing positive for SARS-CoV-2, and similar data for symptomatic children testing negative.

## Methods

### Study design and participants

This prospective cohort study analysed data acquired from voluntary participants in the COVID Symptom Study, who self-reported data through a mobile application (app) launched jointly by Zoe Limited and King's College London on March 24, 2020, freely available to download in the UK (full details in appendix pp 1–2).^10^, ^15^ Briefly, upon registration, participants provide consent for their data to be used in COVID-19 research, and key demographic and comorbidity data (eg, presence of diabetes or asthma). Subsequently, participating individuals are prompted to report daily any symptoms (with direct questions about specific symptoms listed in the appendix p 3, and free-text entry), any SARS-CoV-2 tests and results, vaccination details, and health-care access.^10^ Adult contributors can also proxy-report for others, including children. Relationships between contributor and proxy-reported individuals are not solicited.

Ethics approval was granted by King's College London Ethics Committee (reference LRS-19/201–8210). Data for this study used proxy-reported children (aged 5–17 years), who could not give consent directly. Therefore, consent was from the proxy-reporting adult who was the app user, with governance granted to allow proxy-reported data usage in this circumstance. Full consent details, particularly pertaining to proxy-reported individuals, are provided in the appendix (p 1).

### Procedures

Data from all proxy-reported school-aged children (age 5–17 years) from all four countries of the UK were available from app launch to Feb 22, 2021 (8 weeks after the UK peak SARS-CoV-2 positive specimen date). Data were analysed overall and within two age groups: younger children aged 5–11 years (UK primary school-aged children) and older children aged 12–17 years (UK secondary school-aged children, noting that children can cease education from age 16 years).

Children aged 16–17 years can register and report on the app independently, or be proxy-reported by an adult. However, as described in the appendix (p 2), only proxy-reported 16-year-olds and 17-year-olds were included in this study.

Children were considered symptomatic of SARS-CoV-2 if they were proxy-reported with relevant symptoms^10^, ^16^ between 1 week before and 2 weeks after infection confirmation (either PCR or lateral flow antigen test). Illness duration was calculated from the first symptom (having been previously asymptomatic) until recovery (return to asymptomatic or, if proxy-reporting ceased before becoming asymptomatic, final proxy report). Individuals who were proxy-reported as asymptomatic but subsequently re-reported with symptoms within 1 week of their last symptomatic report were considered unwell from initial presentation (ie, relapsing or remitting illness), with illness duration calculated accordingly. Data from children with reporting gaps longer than 1 week between symptomatic reports were excluded. Individual symptom prevalence and duration were assessed, with duration calculated from first to last report for that symptom.

Symptom burden was calculated as the number of different symptoms reported at least once over defined timeframes (during first week, first 28 days, from day 28 until illness end, and entire illness duration).^2^ Illness with symptoms lasting for 28 days or more was termed LC28 and for 56 days or more was termed LC56. Thus, by virtue of census dates, LC28 could be determined for children whose symptoms commenced on or before Jan 24, 2021, and LC56 for children whose symptoms commenced on or before Dec 29, 2020 (peak positive specimen date). Hospital presentation comprised emergency department presentation or hospital admission following symptom commencement. Proxy-reporting density was defined as the number of episodes of proxy-reporting over illness duration, and proxy-reporting persistence was defined as proxy-reporting until return to asymptomatic.

Several direct symptom questions were added to the app on Nov 4, 2020 (appendix pp 4–5), but these data were not included in the main illness profile analyses.

Free-text reporting was possible across the entire period. Free-text data were divided into themes using descriptive word frequency, and items within themes were independently scrutinised by two clinicians (MAb, ELD) to ensure appropriateness. Individuals reporting symptoms within themes were then counted. Free-text data are reported as descriptive statistics and not included in illness profile analyses (appendix pp 6–8). Free-text data searching included neurological terms and symptoms potentially affecting attention, behaviour, learning or school performance or both; symptoms already assessed by direct questions were excluded.

Illness profiles, including illness duration and symptom burden, were also assessed in children testing negative for SARS-CoV-2, using a randomly selected control cohort (matched 1:1 for age, gender, and week of testing; appendix p 2), and compared with children who tested positive.

Prevalence data for common winter circulating viruses were obtained from national public health databases.^14^

### Outcomes

The primary outcome was illness duration and symptom burden in children who tested positive for SARS-CoV-2 and in matched children who tested negative, assessed overall as well as for younger and older children. Additionally, we assessed individual symptom prevalence and duration, hospital presentation, and the prevalence of prolonged illness duration.

### Statistical analysis

Data are presented using descriptive statistics. Due to rarity (some percentages <5%), CIs were calculated using Poisson distribution. Comparisons of data between groups were done using Wilcoxon signed-rank test, two-tailed χ^2^-tests, or Fisher's exact tests. We used Spearman correlation to assess correlation of illness duration with age. All analyses were done in Python version 3.7.

### Role of the funding source

The funder of the study had no role in study design, data collection, data analysis, data interpretation, or writing of the report.

## Results

**Figure 1:**
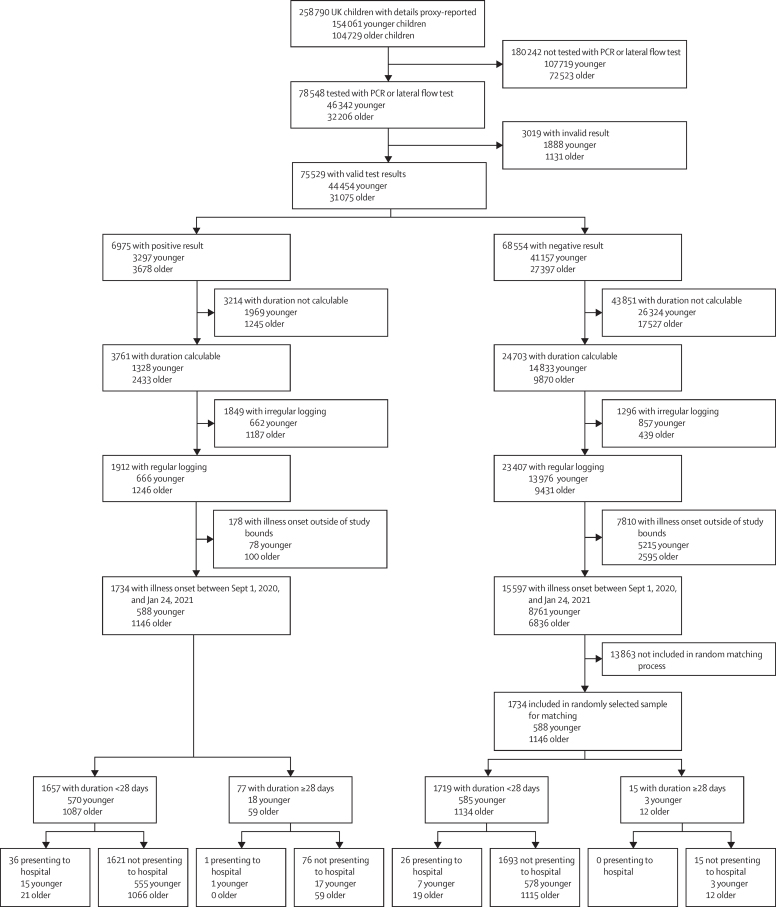
Study flowchart of inclusion and exclusion criteria Overall number for the entire cohort of children is given first. Younger children=aged 5–11 years (UK primary school-aged children). Older children=aged 12–17 years (UK secondary school-aged children). Invalid result=PCR test or lateral flow test result proxy-reported as “failed test” or “still waiting”. Duration calculable=illness onset within defined timeframe of testing for SARS-CoV-2, and with defined endpoint. Regular logging=proxy-reporting at least once every 7 days during illness duration. Irregular logging=proxy-reporting with intervals of more than 7 days between proxy-reports during illness duration. Illness onset outside of study bounds=symptom onset before Sept 1, 2020, or after Jan 24, 2021. Presenting to hospital=either admitted to hospital or seen in the emergency department.

Overall, 258 790 UK children aged 5–17 years were proxy-reported between March 24, 2020, and Feb 22, 2021. Positive SARS-CoV-2 testing was reported in 6975 children, of whom 1912 (666 younger and 1246 older children) had a calculable illness duration and requisite proxy-report logging (figure 1). Because only 36 of these 1912 children had illness onset before Sept 1, 2020 (return-to-school), and given the limited testing access early in the UK pandemic,^13^ analyses were restricted to children with illness onset after Sept 1, 2020. 1734 (588 younger, 1146 older) children were proxy-logged on or before Jan 24, 2021, allowing LC28 to manifest.^2^ Similarly, 1379 (445 younger, 934 older) children had symptoms commencing on or before Dec 29, 2020, allowing LC56 to manifest.^2^ Details regarding the final cohort are presented in the table, and additional demographic details (including ethnicity and index of multiple deprivation decile) are in the appendix (pp 9–10).

**Table:** Characteristics of school-aged children who tested positive for SARS-CoV-2, and the control cohort of children (matched 1:1 for age, gender, and week of testing) who tested negative for SARS-CoV-2

	**Children with positive SARS-CoV-2 test (n=1734)**					**Children with negative SARS-CoV-2 test (matched cohort, n=1734)**
	Younger group (aged 5–11 years, n=588)	Older group (aged 12–17 years, n=1146)	Symptom duration <10 days (n=1183)	Symptom duration ≥28 days (n=77)	Full cohort (n=1734)	
Females	301 (51·2%)	569 (49·7%)	565 (47·8%)	42 (54·5%)	870 (50·2%)	869 (50·1%)
Males	287 (48·8%)	577 (50·3%)	618 (52·2%)	35 (45·5%)	864 (49·8%)	865 (49·9%)
Age, years	9 (7–10)	15 (13–16)	13 (10–15)	14 (12–16)	13 (10–15)	13 (10–15)
Body-mass index (kg/m^2^)	17·0 (15·1–19·7)	20·1 (17·8–22·3)	19·0 (16·5–21·8)	18·6 (16·3–21·8)	19·2 (16·6–21·8)	19·0 (16·5–21·5)
Asthma	69 (11·7%)	147 (12·8%)	134 (11·3%)	10 (13·0%)	216 (12·5%)	229 (13·2%)
Heart disease	1 (0·2%)	1 (0·1%)	2 (0·2%)	0	2 (0·1%)	0
Diabetes	2 (0·3%)	5 (0·4%)	4 (0·3%)	0	7 (0·4%)	6 (0·3%)
Renal disease	0	2 (0·2%)	0	1 (1·3%)	2 (0·1%)	4 (0·2%)
Presentation to hospital	16 (2·7%)	21 (1·8%)	20 (1·7%)	1 (1·3%)	37 (2·1%)	26 (1·5%)
Illness duration, days	5 (2–9)	7 (3–12)	4 (2–6)	46 (32–58)	6 (3–11)	3 (2–7)
Number of symptoms in the first week	3 (2–5)	4 (2–6)	3 (2–5)	6 (4–8)	3 (2–6)	2 (1–4)

Data are n (%) or median (IQR). The cohort of children with positive SARS-CoV-2 testing is presented here both as younger and older groups, and for usual (ie, short) versus extended illness duration. Data refers to children with symptom onset between Sept 1, 2020, and Jan 24, 2021. Common paediatric comorbidities such as neurological or neurodisability disorders (eg, cerebral palsy) were not assessed. Presentation to hospital included presenting to the emergency department or admission to hospital.

Median illness duration in children with COVID-19 was 6 (IQR 3–11) days (table) and was shorter in younger than in older children (5, IQR 2–9 *vs* 7, 3–12) days, (p<0·0001). Age correlated with illness duration (Spearman's rank-order *r*_s_ 0·19, p<0·0001).

**Figure 2:**
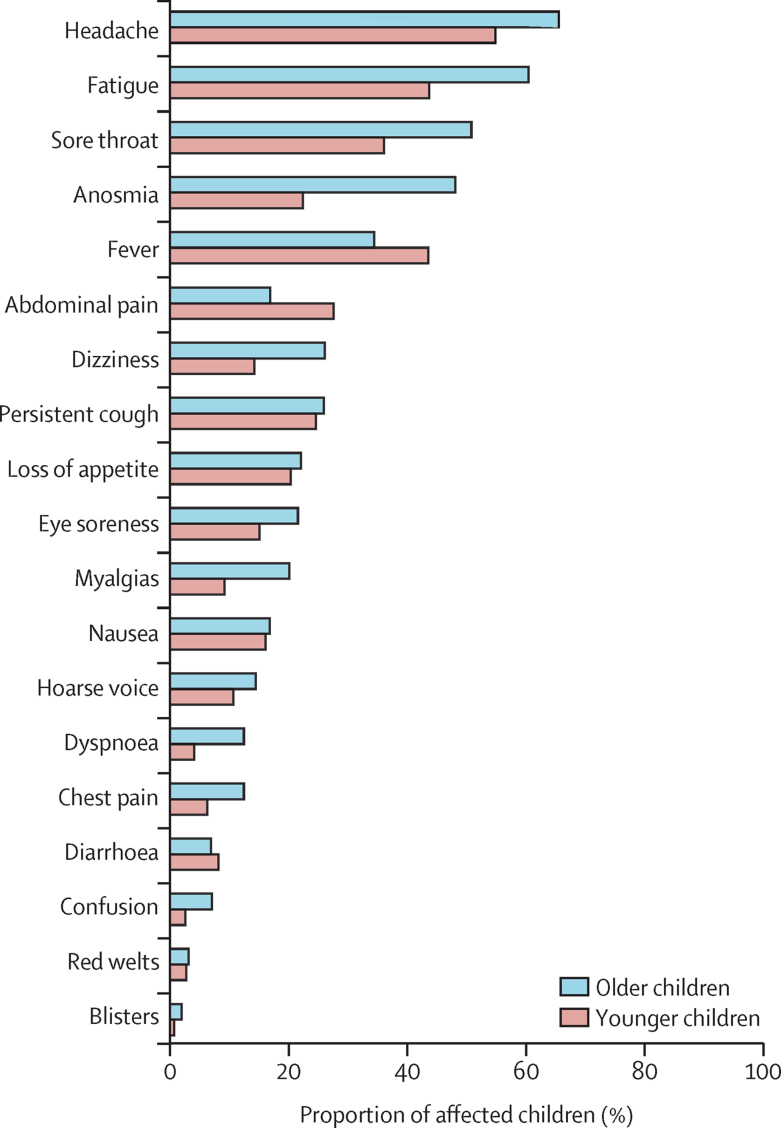
Prevalence of symptoms reported over the course of illness in younger (age 5–11 years, n=588) and older (age 12–17 years, n=1146) children testing positive for SARS-CoV-2 Data refers to children with symptom onset between Sept 1, 2020, and Jan 24, 2021.

**Figure 3:**
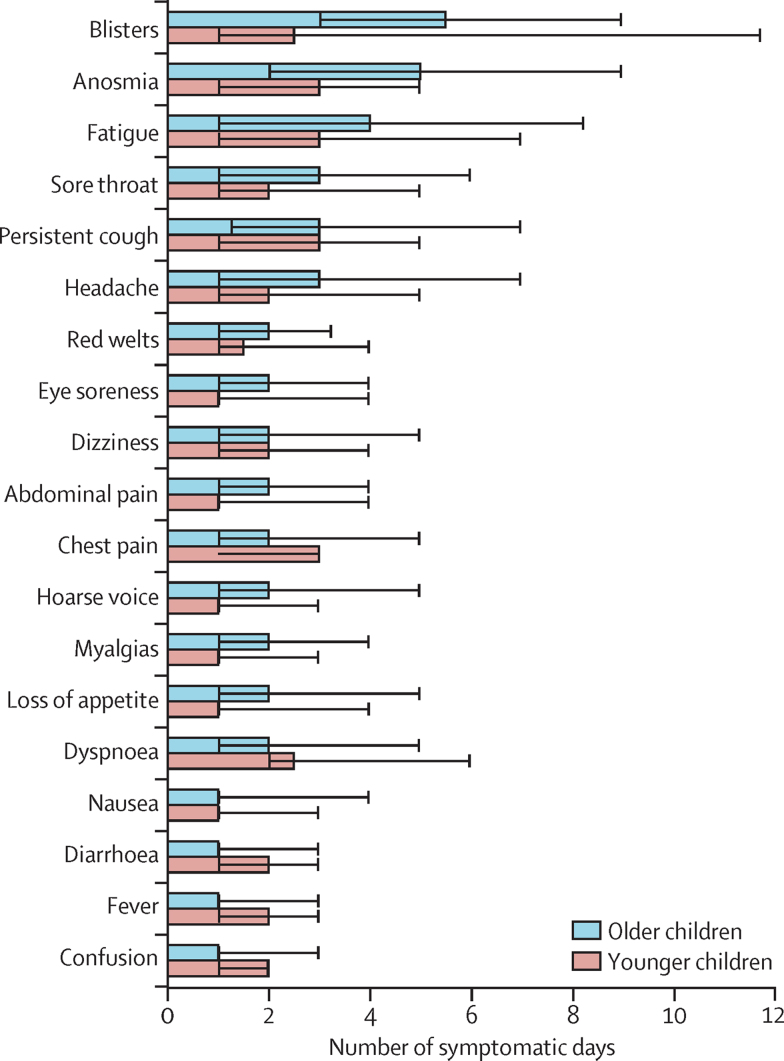
Median duration of each symptom and IQR in younger (age 5–11 years) and older (age 12–17 years) children Data refers to children with symptom onset between Sept 1, 2020, and Jan 24, 2021. Black bars represent IQRs.

Individual symptom prevalence and duration are presented in Figure 2, Figure 3, and the appendix (pp 11–13). The most common symptoms were headache (1079 [62·2%] of 1734 children, of whom 324 [55·1%] of 588 were younger and 755 [65·9%] of 1146 were older children) and fatigue (954 [55·0%] of 1734 children, of whom 258 [43·9%] of 588 were younger and 696 [60·7%] of 1146 were older children). The next most common symptoms in the 588 younger children were fever (257 children [43·7%]), sore throat (213 children [36·2%]), abdominal pain (163 children [27·7%]), and persistent cough (145 children [24·7%]). In the 1146 older children, the next most common symptoms were sore throat (585 children [51·0%]), anosmia (554 children [48·3%]), fever (396 children [34·6%]), and persistent cough (298 children [26·0%]). Overall, 1292 (74·5%) of 1734 children testing positive had fever, cough, anosmia, or a combination of these symptoms.

During the first week of illness, the median symptom burden was 3 symptoms (IQR 2–6) in the whole cohort (3 symptoms [2–5] in younger children and 4 symptoms [2–6] in older children). Symptom distribution by age group and gender is presented in the appendix (pp 12–13).

16 younger and 21 older children with COVID-19 presented to hospital. The symptom profiles of these children were compared with community-managed children, but no statistical comparisons were undertaken, given the low number of hospital attendees (appendix p 18).

**Figure 4:**
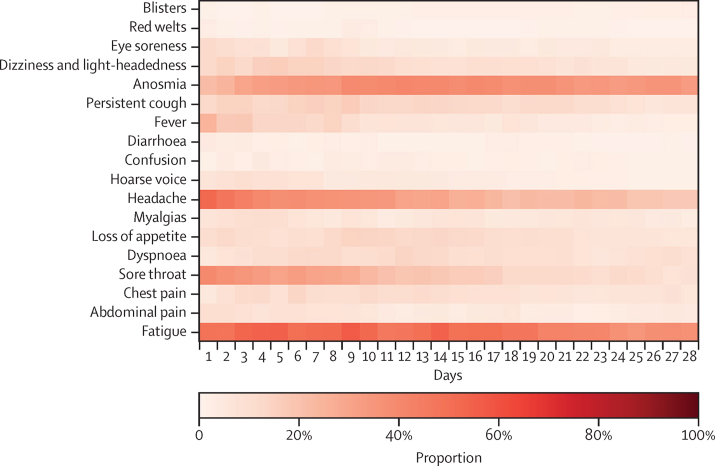
Heat maps showing symptom duration in school-aged children (age 5–17 years) testing positive for SARS-CoV-2 in whom at least one symptom persisted for at least 28 days n=77. Colour bar provides a percentage comparison. Data refers to children with symptom onset between Sept 1, 2020, and Jan 24, 2021.

77 (4·4%, 95% CI 3·5–5·5) of 1734 children had LC28, including 18 younger and 59 older children. The median symptom burden in these children was 6 symptoms (IQR 4–8) during the first week of illness, and 8 symptoms (6–9) over their entire illness. However, after day 28, the median symptom burden was low, at 2 symptoms (IQR 1–4) in the cohort overall (3 symptoms [1–4] in younger children and 1 symptom [1–3] in older children). The most common symptoms experienced by these 77 children over their entire illness were fatigue (65 children [84·4%]), headache (60 children [77·9%]), anosmia (60 children [77·9%]), and sore throat (57 children [74·0%]). The symptom profile and progression over the first 28 days in children with LC28 is presented in figure 4. Headache, fatigue, and sore throat commonly manifest early in illness, with a persistence of fatigue and, to a lesser extent, headache. Anosmia often manifest only later in illness.

25 (1·8%, 95% CI 1·2–2·7) of 1379 children had LC56.^2^ The median symptom burden in these children was 6 symptoms (IQR 4–8) during the first week, and 8 symptoms (6–10) over their entire illness. The most common symptoms in these 25 children over their entire illness were anosmia (21 children [84·0%]), headache (20 children [80·0%]), sore throat (20 children [80·0%]), and fatigue (19 children [76·0%]).

Older children were more likely to manifest LC28 than were younger children (59 [5·1%] of 1146 older children *vs* 18 [3·1%] of 588 younger children; p=0·046). This observation was not evident in children with LC56 (19 [2·0%] of 934 older children *vs* six [1·3%] of 445 younger children; p=0·52).

Regarding symptom reporting from the additional app questions added on Nov 4, 2020 (with a smaller cohort due to a shorter time frame), the most common symptom was rhinorrhoea (198 [39·1%] of 507 younger children and 439 [47·1%] of 932 older children) and then sneezing (143 [28·2%] of 507 younger children and 269 [28·9%] of 932 older children). These were also the most common symptoms observed in children with LC28 over their entire illness, with 33 (52·4%) of 63 children reporting each of rhinorrhoea and sneezing.

Considering potential neurological symptoms other than headache and fatigue, dizziness was reported in 84 (14·3%) of 588 younger children and 300 (26·2%) of 1146 older children, with a median duration of 2 days (IQR 1–4) for younger children and 2 days (1–5) for older children. Confusion (including disorientation and drowsiness) was reported in 15 (2·6%) of 588 younger children and 81 (7·1%) of 1146 older children, with a median duration 2 days (IQR 1–2) for younger children and 1 day (1–3) for older children. No statistical comparisons of these symptoms in children with shorter or longer illness duration were made, given their low prevalence in children with LC28 (figure 4).

Considering the questions added on Nov 4, 2020, the experience termed brain fog was reported for 30 (5·9%) of 507 younger children and 105 (11·3%) of 932 older children, with a median duration of 1 day (IQR 1–4) for younger children and 2 days (1–5) for older children. Low mood was reported for 40 (7·9%) of 507 younger children and 145 (15·6%) of 932 older children, with a median duration of 2 days (IQR 1–3·5) for younger children and 2 days (1–4) for older children (appendix p 17).

Free-text searching for specific neurological symptoms disclosed few reports of weakness (two children) or tics (one child). No severe neurological manifestations were reported (appendix pp 6–8). Irritability (three children, two irritable and one grumpy), emotional difficulties (two children, one with emotional difficulty and one with low mood), and behavioural difficulties (one child) were reported rarely; impaired attention and concentration were not reported.

15 597 symptomatic children (including 8761 younger and 6836 older children) testing negative for SARS-CoV-2 were proxy-reported at least once weekly within the study period and had calculable illness duration. Of these, 1734 were matched to children testing positive (table; appendix pp 9–10). Their median illness duration was 3 days (IQR 2–7) overall (3 days [2–7] for younger children and 4 days [2–7] for older children), shorter than for children testing positive (Wilcoxon test, p<0·0001)Individual symptom duration and symptom prevalence are presented in the appendix (pp 14, 19). The most common symptoms over their entire illness were as follows: in the 588 younger children, sore throat (274 children [46·6%]), headache (228 children [38·8%]), fever (179 children [30·4%]), fatigue (158 children [26·9%]), and abdominal pain (145 children [24·7%]); in the 1146 older children, sore throat (695 children [60·6%]), headache (559 children [48·8%]), fatigue (426 children [37·2%]), fever (234 children [20·4%]), and persistent cough (236 children [20·6%]). Overall, 805 (46·4%) of 1734 children testing negative had fever, cough, anosmia, or a combination of these symptoms.

Few children testing negative had an illness duration of 28 days or more (15 of 1734 children, 0·9%, 95% CI 0·5–1·4), which was lower than for children testing positive overall (p<0·0001) and within age groups (younger children, three [<1%] of 588 *vs* 18 [3·1%] of 588; p=0·0010; older children, 12 [1·0%] of 1146 *vs* 59 [5·1%] of 1146; p<0·0001). The symptom profile of these children over their first 28 days of illness is shown in the appendix (p 20). These children had a greater symptom burden than did children who tested positive for SARS-CoV-2 who had illness duration for 28 days or longer, both in the period beyond 28 days (5 symptoms [IQR 1·5–6·5] *vs* 2 symptoms [1–4]; p=0·0050; appendix p 22). and over their entire illness duration (9 symptoms [IQR 7·5–11] *vs* 8 symptoms [6–9]; p=0·025; appendix p 23).

Proxy-reporting was assiduous for all children. Proxy-reporting density was higher in children testing negative for SARS-CoV-2 than for children testing positive (younger, 1·0, IQR 0·7–1·0 *vs* 0·9, 0·6–1·0; older, 1·0, 0·7–1·0 *vs* 0·8, 0·6–1·0), noting the shorter illness duration in children testing negative. Proxy-reporting until asymptomatic (ie, perseverance) was higher in children testing negative than in children testing positive (1674 [96·5%] of 1734 *vs* 1551 [89·4%] of 1734; p<0·0001).

Among children testing positive, symptom logging ceased in 183 children before an asymptomatic report (22 with illness duration ≥28 days, 161 with duration <28 days). In the 77 children with illness duration of 28 days or more, proxy-reporting continued until an asymptomatic report was received in 55 (71·4%). However, the remaining 22 children already had symptoms for at least 28 days and thus fulfil the definition of LC28. In children with illness duration of less than 28 days, an asymptomatic report was received for 90·3% (1496 of 1657). For the remaining 161 children, the median symptom burden at last report was 2 symptoms (IQR 1–3), and proxy-reporting usually ceased early in illness (in 11·3% of children with illness duration of <10 days, and 5·5% of children with illness duration of ≥10 days). Thus, we assumed that proxy-reporting cessation corresponded to illness resolution (ie, the child had recovered) and we calculated illness duration accordingly.

We considered the effect of this assumption. Excluding all children testing positive for whom a healthy report was not logged, the median illness duration in the remaining 1551 children was unchanged (6 days, IQR 3–11 days) with LC28 prevalence of 3·5% (55 of 1551), within the previously calculated confidence interval of 3·5–5·5, using data from the entire cohort. Excluding only the 161 children with assumed short-term symptoms, but including all children with LC28 (regardless of receipt of healthy report), LC28 prevalence was 4·9% (77 of 1573), again within the previously calculated confidence interval of 3·5–5·5.

In children testing negative, logging ceased in 60 children before a healthy report (four of 15 children with long illness duration and 56 of 1719 children with assumed short illness duration).

## Discussion

In this study, we show that symptomatic SARS-CoV-2 infection in UK children aged 5–17 years is usually of short duration (6 days *vs* 11 days in adults^2^), with low symptom burden. Prolonged illness can occur but is infrequent (4·4% for LC28 and 1·8% for LC56) and lower than for adults (13·3% for LC 28 and 4·5% for LC56).^2^ We found age to correlate with illness duration overall and in children with illness duration of 28 days or more, consistent with our previous findings in adults.^2^

The most common symptoms in our cohort of children with COVID-19 were headache (62·2%) and fatigue (55·0%). A meta-analysis of studies that included both community-based children and those admitted to hospital identified fever (47%) and cough (42%) as the most common symptoms in children with COVID-19; however, headache and fatigue were only assessed in half of the contributing cohorts.^3^ In our cohort, 37·7% reported fever and 25·5% reported a persistent cough. Anosmia and dysosmia were common (39·6%), which was higher than in a small study of adolescents with mild to moderate COVID-19 in which anosmia was reported in 24·1% of participants,^17^ noting that anosmia was a core symptom for testing access during our study period. Relevant to UK testing criteria, anosmia became more prevalent later in illness, at least in children with LC28.

In children with LC28, by day 28 the symptom burden was low (median 2 symptoms). Fatigue was proxy-reported at some stage in most participants (84·4%). In our adult study, fatigue was almost universal in LC28 (97·7% at some stage during illness);^2^ other adult studies have reported persistence of fatigue as 53·1% at 60 days^18^ and 52·3% at 10 weeks.^19^

Few epidemiological studies provide normative data for headache and fatigue in children. Regarding headache, a systematic review of population-based studies in individuals younger than 20 years reported that about 60% were prone to headache;^20^ and 66% of children aged 5–15 years reported headaches over the previous year.^21^ Regarding fatigue, a study of 2936 children found that 4·4% had “more than a few days of disabling fatigue”.^22^ In one study, prevalence of chronic fatigue syndrome (here defined as “disabling fatigue lasting more than 3 months…with no other cause”) was 1% in children aged 11–16 years;^23^ and in the Avon Longitudinal Study prevalence of “chronic disabling fatigue” (here defined as “fatigue lasting more than 6 months and associated with absence from full-time school or had prevented...activities”) was 1·5% in children aged 13 years and 2·2% in children aged 16 years.^24^ Considering fatigue after viral infection, median illness duration after Epstein–Barr virus infection in symptomatic university students was 10 days, with a median duration of fatigue of 15·5 days.^25^

Being able to contextualise COVID-19 in children is complicated by a lack of contemporaneous data on illness profiles after other viral infections. A study of 242 158 children and adolescents with COVID-19 (9769 admitted to hospital) and 2 084 180 children with influenza during 2017–19 (numbers admitted to hospital unclear) suggested that dyspnoea, anosmia, and gastrointestinal tract symptoms were more common with COVID-19 than with influenza.^26^ However, symptoms were only reported as present or absent at 30 days in both groups, preventing more granular comparisons.

A strength of our study is its comparison of contemporaneous illness profiles of symptomatic children testing positive versus negative for SARS-CoV-2. Children testing positive had longer median illness duration (6 days *vs* 3 days) and were more likely to have illness duration of at least 28 days (4·4% *vs* 0·9%). However, some children testing negative also had illness duration of at least 28 days; these children had higher symptom counts over their entire illness duration and at day 28, acknowledging our small sample size. We considered whether some of these children might have false-negative results. However, there is no evidence that the sensitivity and specificity of SARS-CoV-2 testing differ in children compared with adults, with sensitivity for PCR tests for SARS-CoV-2 of about 95%. The symptom profiles of children testing positive and negative suggest some differences, although these were not statistically assessed. Notably, the prevalence of non-SARS-CoV-2 respiratory viruses (influenza A, influenza B, parainfluenza, adenovirus, and respiratory syncytial virus) were unusually low over the 2020–21 winter in the UK,^14^, ^27^, ^28^ except for the rhinovirus peak commonly observed in September (return-to-school).^14^ With the relaxation of social distancing in the UK, these illnesses might return towards their usual higher prevalences. Our data emphasise that other childhood illnesses might also have protracted burdensome courses, requiring consideration in post-pandemic service planning.

Short-term and long-term effects of COVID-19 on school performance and learning have been a matter of concern.^11^ In our cohort, attentional problems, memory complaints, and anxiety were not reported, and cases of low mood and irritability were consistent with previous school-aged population data. Our data do not support anecdotal reports of weakness and seizures as being common in children with COVID-19 of any duration, and no severe neurological symptoms were reported. However, any persistent illness can have adverse mental health outcomes and affect school attendance.^29^ Mental health data could not be proxy-reported, limiting our ability to assess mental health comprehensively in children during the pandemic, and we did not collect data regarding school attendance.

In considering other data sources, the UK Office for National Statistics (ONS) did SARS-CoV-2 testing irrespective of symptoms from Dec 2 to Dec 10, 2020, in 7089 pupils drawn from 121 schools (41 primary, 80 secondary); 0·94% of pupils and 0·99% of staff in primary schools tested positive, and 1·22% of pupils and 1·64% of staff in secondary schools. However, ONS have warned that their conclusions might not have general validity, because there was deliberate oversampling of schools with higher infection rates early after return-to-school. Moreover, these figures do not capture fluctuations as the pandemic progressed. In our integrated data across the pandemic, 6975 (2·7%) of 258 790 proxy-reported children tested positive. It should be noted that PCR testing was only available for symptomatic individuals (and rarely during the first wave); and proxy-reporting was voluntary and through the COVID Symptom Study app with its 4·6 million adult user base over-representative of women, those of White background, and those with above-average socioeconomic status^12^ compared with the general UK population. Furthermore, we cannot characterise regional variability because geographic information was unavailable for many participants. These issues might cause selection bias in our study.

Reporting of SARS-CoV-2 data is not uniform across the UK: the four countries report data for children and young people within differing timeframes and age groups (appendix p 15). Conservatively, our study represents about 1–2% of UK school-aged children testing positive during the pandemic.

The most recent ONS data (released April, 2021) estimated that 9·8% of children aged 2–11 years and 13·0% of children aged 12–16 years had ongoing symptoms 5 weeks after testing positive, with 7·4% of those aged 2–11 years and 8·2% of those aged 12–16 years still reporting symptoms at 12 weeks.^30^ These figures decreased compared with previous ONS estimates in January, 2021 (eg, from 12·9% to 9·8% in children aged 2–11 years).^30^, ^31^ The ONS reported a control group (never symptomatic, never tested, never self-isolated, and never a contact of anyone testing positive) with baseline pooled symptom rates of 2% in children aged 2–11 years and 1·7% in children aged 12–16 years.^30^, ^31^ There is limited published detail of ONS methodology.

The contrast between our LC28 prevalence and ONS figures might result from ONS requiring two consecutive asymptomatic visits to define illness end; thus, children with asymptomatic periods of more than 1 week between symptoms would be captured by ONS but not by the present study. Notably, ONS's sensitivity analysis of the effect of defining illness end to a single asymptomatic visit markedly lowered their prevalence estimates, especially for illness of more than 12 weeks (from 13·7% to 0·9%^30^), which is more consistent with our results. Additionally, ONS estimates are made using current and recalled data collected in the first week of each month, whereas our app-based data are collected in real-time. Our data concord with a small Australian study reporting follow-up of 151 children with SARS-CoV-2 (median age 3 years): 12 had symptoms 3–8 weeks after initial presentation (most commonly cough or fatigue, or both), and all returned to baseline health by 3–6 months.^32^

Our study is one of the largest UK citizen-collaborative epidemiological studies.^2^, ^15^ Our census points allowed capturing of all children with illness duration of at least 8 weeks who presented before the UK peak positive specimen date. By ensuring that symptoms concorded with testing, we could attribute symptoms to SARS-CoV-2 infection defined by contemporaneous test results. We avoided bias from limited test availability early in the pandemic by restricting analyses from Sept 1, 2020, but we acknowledge there were still some access issues.^33^ Specifically, to be eligible for PCR testing, individuals were required to have fever, cough, anosmia, or a combination of these symptoms, criteria that were largely informed by adult symptomatology, which might miss some paediatric manifestations of COVID-19 (eg, abdominal pain^3^, reported in 27·8% of our younger children). Free-text data did not suggest common symptoms unique to children; qualitative analysis was not undertaken given its ad hoc collection and potential bias from additional direct questions after Nov 4, 2020 (ie, once directly asked, a symptom was unlikely to be free-text reported). Additionally, we did not ask specifically about MIS-C. Only 74·5% of children testing positive and 46·4% of children testing negative were reported to have fever, cough, anosmia, or a combination of these symptoms. We do not know why the remaining children were tested. The positive predictive value of any symptom varies according to illness prevalence, and here is clearly subject to the pandemic dynamics.^34^ However, nearly a quarter of symptomatic children testing positive for SARS-CoV-2 during the UK's second wave did not report any of these symptoms, suggesting that UK testing policy needs reconsideration.

We also acknowledge that symptoms were proxy-reported rather than directly ascertained. This is common in clinical assessment of children, particularly younger children. We do not have linkage to general practice or hospital records to validate proxy-reported data. Crucially, proxy-reported children depended upon an adult with access and capacity to participate in the COVID Symptom Study.^10^ Socioeconomic demographics of our study's participants, along with UK general population data, are presented in the appendix (pp 9–10); beyond this we cannot comment on the characteristics of proxy-reported versus non-reported children. We did not have information on the relationship of the contributor to the proxy-reported child, which could influence reporting. For example, an unwell contributor might be too ill to proxy-report for a child; however, our high proxy-reporting density and perseverance of all symptomatic children suggest that this was uncommon. Current or previous symptoms experienced by contributors might also influence their proxy-reporting.

Our national study provides the first detailed description of COVID-19 in symptomatic school-aged children. Although uncommon, a small proportion of children have prolonged illness duration and persistent symptoms. Our LC56 data provide reassurance regarding their long-term outcomes. The symptom burden in children testing negative for SARS-CoV-2 but with long illness duration emphasises that allocation of appropriate resources will be necessary for any child with prolonged illness, whether from SARS-CoV-2 infection or other illness. Our study provides timely and crucial data about the effect and implications of the pandemic on UK paediatric health-care resource allocation.

**This online publication has been corrected. The corrected version first appeared at thelancet.com/child-adolescent on August 31, 2021**

## Data sharing statement

Data collected in the COVID Symptom Study smartphone app can be shared with other health researchers through the UK National Health Service-funded Health Data Research UK and Secure Anonymised Information Linkage consortium, housed in the UK Secure Research Platform (Swansea, UK). Anonymised data are available to be shared with researchers according to their protocols in the public interest https://web.www.healthdatagateway.org/dataset/594cfe55-96e3-45ff-874c-2c0006eeb881.

## Declaration of interests

CH, SS, KR, and JCP are employees of Zoe Limited. TDS reports being a consultant for Zoe Limited, during the conduct of the study. All other authors declare no competing interests.
