# Correction to *Lancet Child Adolesc Health* 2021; 5: 708–18

**DOI:** 10.1016/S2352-4642(21)00276-5

**Published:** 2021-10

*Molteni E, Sudre CH, Canas LS, et al. Illness duration and symptom profile in symptomatic UK school-aged children tested for SARS-CoV-2.* Lancet Child Adolesc Health *2021; published online Aug 3. https://doi.org/10.1016/S2352-4642(21)00198-X*—In this Article, a date in the Summary was incorrect. The correct sentence now reads: 1734 children (588 younger and 1146 older children) had a positive SARS-CoV-2 test result and calculable illness duration within the study timeframe (illness onset between Sept 1, 2020, and Jan 24, 2021). This correction has been made to the online version as of Aug 31, 2021, and the printed version is correct.

