# Supplementary material for: Illness duration and symptom profile in symptomatic UK school-aged children tested for SARS-CoV-2
Source: Lancet Child Adolesc Health. 2021 Oct;5(10):708–18. doi: 10.1016/S2352-4642(21)00198-X (PMC8443448; doi:10.1016/S2352-4642(21)00198-X)

THE LANCET
Child & Adolescent Health

Supplementary appendix

This appendix formed part of the original submission and has been peer reviewed. We post it as supplied by the authors.

Supplement to: Molteni E, Sudre CH, Canas LS, et al. Illness duration and symptom profile in symptomatic UK school-aged children tested for SARS-CoV-2. *Lancet Child Adolesc Health* 2021; published online Aug 3. http://dx.doi.org/10.1016/S2352-4642(21)00198-X.

SUPPLEMENTARY DOCUMENT

Illness duration and symptom profile in symptomatic UK school-aged children tested for SARS-CoV-2

Erika Molteni, Carole H. Sudre, Liane S. Canas, Sunil S. Bhopal, Robert C. Hughes, Michela Antonelli, Benjamin Murray, Kerstin Kläser, Eric Kerfoot, Liyuan Chen, Jie Deng, Christina Hu, Somesh Selvachandran, Kenneth Read, Joan Capdevila Pujol, Prof Alexander Hammers, Prof Tim D. Spector, Prof Sebastien Ourselin, Claire J. Steves, Marc Modat, Michael Absoud, Prof Emma L. Duncan.

Supplementary Methods.

The ZOE-CSS mobile application and usage

The CSS study is undertaken through a mobile application for iPhone® and Android® users launched jointly by Zoe Limited. and KCL on 24 March 2020, and released in UK, USA and Sweden.[10] Smartphone ownership in the UK is extensive, with little evidence this varies greatly across socio-economic groups. Software was tested before app launch, and before release of any version update. Software for repeatable and consistent data extraction, curation, and analytics was also engineered (http://arxiv.org/abs/2011.00867).[15]

At time of registration, all participants provide consent for their data to be used for research. Participants can withdraw from the study at any time, with their data subsequently excluded from analysis.

Upon registration, participants provide key demographic (age, weight, height, geographic area, occupation [health care worker or not]) and comorbidity data (e.g. diabetes, cardiovascular disease, asthma). Participating individuals are then prompted to report daily. Multiple daily reports are allowed, to capture new and/or evolving symptoms. Self-reporting adults could also answer specific questions regarding mental health.

Control cohort characterisation

We randomly selected a control cohort among the negatives, matched 1:1 for age, gender, and week of testing with the children testing positive. Specifically, for each child testing positive for SARS-CoV-2, a corresponding control from the group of children testing negative for SARS-CoV-2 was selected on the basis of a minimum Euclidean distance based on age, gender, and week of testing. If more than one candidate child was identified within the control children (i.e., multiple children with the same minimal distance value), one was randomly selected from the pool of candidates.

Data from children aged 16-17 years

Children aged 16-17 years can register and report on the app independently, or be proxy-reported by an adult. In the total cohort of 16- and 17-year-old individuals, 29,047 self-logged data (447 reporting testing positive) compared to 32,271 proxy-reported (1,197 reported as testing positive). Illness duration could only be calculated in ten self-logged versus 381 proxy-logged 16- and 17-year-olds; and concurrent self-reporting and proxy-reporting could not be excluded. Thus, we included data from proxy-reported 16- and 17-year-olds only in our analyses.

Supplementary Table 1. List of symptom questions asked by the COVID Symptom Study app at 1 September 2020. Answers were yes/no, unless indicated otherwise.

Symptom	COVID Symptom Study app question
**Fever**	Do you have a fever?
**Persistent Cough**	Do you have a persistent cough (coughing a lot for more than an hour, or 3 or more coughing episodes in 24 hours)?
**Fatigue**	Are you experiencing unusual fatigue? (no; mild fatigue; severe fatigue/ I struggle to get out of bed)
**Dyspnoea**	Are you experiencing unusual shortness of breath? (no; yes mild symptoms/ slight shortness of breath during ordinary activity: yes significant symptoms - breathing is comfortable only at rest; yes, severe symptoms/ breathing is difficult even at rest).
**Anosmia**	Do you have a loss of smell/taste?
**Hoarse Voice**	Do you have an unusually hoarse voice?
**Chest Pain**	Are you feeling an unusual chest pain or tightness in your chest?
**Abdominal Pain**	Do you have an unusual abdominal pain?
**Diarrhoea**	Are you experiencing diarrhoea?
**Headache**	Do you have a headache? How often are you experiencing headaches? (number)
**Confusion**	Do you have any of the following symptoms: confusion, disorientation or drowsiness?
**Eye Soreness**	Do your eyes have any unusual eye-soreness or discomfort (e.g., light sensitivity, excessive tears, or pink/red eye)?
**Loss of Appetite**	Have you been skipping meals?
**Nausea**	Have you felt nauseous or experienced vomiting?
**Dizziness**	Are you experiencing dizziness or light-headedness?
**Sore Throat**	Do you have a sore throat?
**Myalgias**	Do you have unusual strong muscle pains?
**Red Welts**	Have you had raised, red, itchy welts on the skin or sudden swelling of the face or lips?
**Blisters**	Have you had any red/purple sores or blisters on your feet, including your toes?

Supplementary Table 2. List of symptom questions asked by the COVID Symptom Study app after changes implemented on 4 November 2020. Questions were: Do you have (symptom)? Answers were yes/no, unless indicated otherwise. New symptoms added on 4 November 2020 are indicated in bold.

**Symptom**	**COVID Symptom Study app question**
Fever	Fever (at least 37.8C or 100F)
Persistent Cough	Persistent cough (coughing a lot for more than an hour or 3 or more coughing episodes in 24 hours)
Fatigue	Unusual fatigue… (no; mild fatigue; severe fatigue/ I struggle to get out of bed)
Dyspnoea	Shortness of breath or trouble breathing (no; yes mild symptoms/ slight shortness of breath during ordinary activity: yes significant symptoms/ breathing is comfortable only at rest; yes, severe symptoms/ breathing is difficult even at rest).
Anosmia/**Ageusia**	Loss of smell / taste
Hoarse Voice	Unusually hoarse voice
Chest Pain	Unusual chest pain or tightness in your chest
Abdominal Pain	Unusual abdominal pain or stomach-ache
Diarrhoea	Diarrhoea
**Stools**	How many loose stools in the last 24 hours?
**Headache Frequency**	How often are you experiencing headaches?
Confusion	Confusion, disorientation or drowsiness
Eye Soreness	Do your eyes have any unusual eye-soreness or discomfort (e.g. light sensitivity, excessive tears, or pink/red eye)?
Loss of appetite	Skipping meals
Headache	Headache
Nausea	Nausea or vomiting
Dizziness	Dizziness or light-headedness
Sore Throat	Sore or painful throat
Myalgias	Unusual strong muscle pains or aches
Red Welts	Raised, red, itchy welts on the skin or sudden swelling of the face or lips
Blisters	Red/purple sores or blisters on your feet, including your toes

**Allergy Exacerbation**	Increase in your usual allergy symptoms
**Rashes**	Rash on your arms or torso
**Sensitive Skin**	Strange, unpleasant sensations in your skin like pins & needles or burning
**Hair Loss**	Unusual hair loss
**Low Mood**	Feeling down, depressed or hopeless
**Brain Fog**	Loss of concentration or memory (brain fog)
**Dysosmia/Dysgeusia**	Altered smell / taste (things smell or taste different to usual)
**Rhinorrhoea**	Runny nose
**Sneezing**	Sneezing more than usual
**Ear Pain**	Earache
**Tinnitus**	Ringing in your ears
**Lymphadenopathy**	Swollen neck glands
**Palpitations**	Unusually fast or irregular heartbeat (palpitations)
**Arthralgias**	Unusual joint pains or aches
**Mouth Ulcers**	Mouth or tongue ulcers
**Tongue Changes**	Changes to tongue surface

Supplementary Results.

Supplementary Table 3. Theme summary from the free-text scrutiny.

Twelve themes were identified and ordered by text word frequency. For each theme, numbers of children (overall and within younger/older age groups) are charted, with keywords used in the automatic search (asterisk at the end of search keywords indicates free term termination (wildcard)) and the number of children for whom each simple or combined term was reported. Free-text was also searched for specific terms of interest, including neuromuscular symptoms (e.g., weakness, paralysis, tics, seizure), and symptoms potentially affecting attention, learning, and school performance (e.g., anxiety, irritability). Summation of the number of children with each symptom within a theme may not equal the number of children within the theme overall, due to multiple terms and negations in free-text. Data on number of individuals reporting a term refers to children with symptom onset between 1 September 2020 and 24 January 2021.

Theme number	Theme name	Number overall	Number of younger children	Number of older children	Search keywords	Numbers of individuals reporting a term
1	Respiratory tract symptoms	131	41	90	nose, sinus, chest, congest*, cold, sneez*, wheez*, throat, tonsillitis, asthma, phlegm, mucus, coughing blood	cold (46), blocked nose, blocked sinuses (17), stuffy nose (5), sinus pain, burning sinuses, sore nose, nose pain (18), congestion (12), burning nose (3), clearing throat (7), dry throat (1), tickle in throat, irritated throat, itchy throat (6), tonsillitis (5), headcold (1), sneezing (2), nose bleeding (2), mucus (8), phlegm (11), fleming (2), asthma (8), wheeze (1), quick breath (1), tight chest (2), burning sensation in chest (1), "pins and needles" in chest (1).

2	Cutaneous manifestations	42	18	24	skin, rash, eczema, impetigo, pale, itch*, spot, finger	eczema (7), rash (5), body rash (1), rash on torso (3), rash on hands (1), rash on face (7), rash on elbows (1), sensitive skin (4), itchy skin (9), itchy bumps on toe skin, toe lumps (2), dry skin (2), red spots (3), white spots on legs (1), finger chilblains (3), pale (4), chilblain on finger knuckle (1).
3	Oral cavity manifestations	29	11	18	mouth, tongue, lip, throat ulcer	mouth ulcers (6), sore or sensitive mouth (4), tongue rash (3), tied tongue and difficulty in speaking (1), white or purple spots on tongue (2), white tongue (1), dry mouth (1), dry lips (1), swollen lips (1), burning or sore lips (3), rash around the lips (1).
4	Joint symptoms and lymphadenopathy	23	9	14	gland, sore	swollen glands (3), sore legs or hip (2), sore back (1), sore joints (1), sore neck glands (1).
5	Ocular symptoms	16	2	14	eye, vision, teary	aching or sore eyes (1), itchy eyes (1), pain behind the eyes (1), eye heaviness or discomfort (3), blurred vision (2), double vision from one eye (2), seeing purple (2), twitchy eye (1), teary eyes (1).
6	Cardiovascular/autonomic symptoms	14	4	10	sweaty, faint, flush, palpitations, heartbeat, shak*	shaky (2), faint (2), palpitations (1), rapid intermittent heartbeat (1), flushed (1), sweaty (1).
7	Sleep disturbance	13	3	10	sleep, insomnia	difficulty or problems in sleeping (5), disturbed or broken sleep (5), unusual moaning in sleep (1), day sleepiness (1), unable to sleep (1).

8	Otological symptoms	7	1	6	ear	hearing loss (1), ringing or popping in ears (2), blocked ear (1), itchy ear (1), glue ear (1).
9	Mental, mood and affective health	6	4	2	concentration, attention, focus, irritab*, emotion, grumpy, behav*, mood, anxiety	irritability (2), low mood (1), "very grumpy" (1), emotional (1), odd behaviour (1).
10	Neurological	6	1	5	tic, twitch, weak*, paralysis, balance, ataxia, walk*, seizure, convulsions, paroxysm, sensory, fit	physical tics (1), weakness (2).
11	Genitourinary symptoms	5	3	2	urin*, bladder, kidney, penis, genital itching	frequent need to urinate (1), urinary tract infection (2), penis infection (1).
12	Gastrointestinal symptoms	3	1	2	constipation, stomach	stomach discomfort (2).
13	Miscellaneous	17	6	11	jaw, thirst, swelling, sugar, toe	swelling (2), low blood sugar with no diabetes (1), increased thirst (2), mottled toes (1), toe swelling (1), toe lumps (1).

Supplementary Table 4. Additional demographic data for proxy-reported children considered for the study.

Data refers to children with symptom onset between 1 September 2020 and 24 January 2021. Geographical location was available for many but not all contributors, as indicated. Age, BMI and IMD are indicated in median and quartiles [first;third]. BMI, body mass index. BAME, Black, Asian and Minority Ethnicity groups.

	**All contributors by proxy** (n=258,790)	**Sample tested for SARS-CoV-2** (n=78,548)	**Sample tested positive for SARS-CoV-2** (n=6,975)	**Sample tested positive for SARS-CoV-2 and included in the symptom study** (n=1,734)	**Sample tested negative for SARS-CoV-2** (n=68,554)	**Sample tested negative for SARS-CoV-2 and included in the symptom study after 1:1 match** (n=1,734)	**Whole UK**
**Age** (years)	10 [6-14]	10 [6-14]	12 [8-15]	13 [10-15]	10 [6-14]	13 [10-15]	10.8
**BMI**	18.26 [15.74-21.52]	18.03 [15.65-21.19]	18.78 [16.14-21.75]	19.22 [16.61-21.77]	17.96 [15.62-21.09]	18.98 [16.53-21.50]	
**Ethnicity**							
White	235,841	71,257	6,238	1,572	62,288	1,613	6,810,455 (80.4%)
BAME	22,055	7,071	714	158	6,081	116	1,663,162 (19.6%)
Prefer not to say	894	220	23	4	185	5	NR

**Location** (available data)	212,359	66,367	6,141	1,460	57,630	1,436	
England	188,730	61,806	5,738	1,380	53,754	1,359	8,723,931
Wales	11,980	2,344	258	55	1,906	38	464,397
Scotland	10,248	1,899	114	22	1,699	35	757,447
Northern Ireland	1,401	318	31	3	271	4	320,336
**Index of Multiple Deprivation Decile (1: least deprived, 10: most deprived)**	7 [5-9]	7 [5-9]	7 [5-9]	8 [5-9]	7 [5-9]	8 [5-9]	5.5

Supplementary Table 5. Symptoms over the entire illness duration in children testing positive for SARS-CoV-2. Data refers to children with symptom onset between 1 September 2020 and 24 January 2021.

	**Sample tested positive for SARS-CoV-2**		
	**Younger children (aged 5-11 years, n=588)**	**Older children (aged 12-17 years, n=1,146)**	**Overall cohort (n=1,734)**
Headache	324	755	1,079 (62.2%)
Fatigue	258	696	954 (55.0%)
Sore Throat	213	585	798 (46.0%)
Anosmia	132	554	686 (39.6%)
Fever	257	396	653 (37.7%)
Abdominal Pain	163	194	357 (20.6%)
Dizziness	84	300	384 (22.1%)
Persistent Cough	145	298	443 (25.5%)
Loss of Appetite	120	254	374 (21.6%)
Eye Soreness	89	248	337 (19.4%)
Myalgias	54	231	285 (16.4%)
Nausea	95	193	288 (16.6%)
Hoarse voice	63	166	229 (13.2%)
Chest Pain	37	143	180 (10.4%)
Dyspnoea	24	143	167 (9.6%)
Diarrhoea	48	79	127 (7.3%)
Confusion	15	81	96 (5.5%)
Red Welts	16	36	52 (3.0%)
Blisters	4	22	26 (1.5%)

Supplementary Table 6. Symptoms by age group (younger: 5-11 years; older: 12-17 years), gender, and test status, considered over the entire illness duration. Data refers to children with symptom onset between 1 September 2020 and 24 January 2021.

	**Sample tested positive for SARS-CoV-2**						**Sample tested negative for SARS-CoV-2**					
	**Younger children (n=588)**		**Older children (n=1,146)**		**Overall cohort (n=1,734)**		**Younger children (n=588)**		**Older children (n=1,146)**		**Overall cohort (n=1,734)**	
**Gender**	Female	Male	Female	Male	Female	Male	Female	Male	Female	Male	Female	Male
Fatigue	34%	34%	34%	34%	56%	54%	27%	27%	37%	37%	34%	34%
Abdominal pain	19%	18%	19%	18%	24%	17%	27%	23%	15%	15%	19%	18%
Chest pain	8%	7%	8%	7%	12%	9%	4%	3%	10%	8%	8%	7%
Sore throat	58%	53%	58%	53%	50%	42%	50%	43%	63%	58%	58%	53%
Dyspnoea	5%	6%	5%	6%	12%	7%	3%	4%	6%	6%	5%	6%
Loss of Appetite	12%	12%	12%	12%	23%	20%	11%	11%	13%	13%	12%	12%
Myalgias	9%	8%	9%	8%	17%	16%	5%	7%	11%	9%	9%	8%
Headache	48%	43%	48%	43%	65%	59%	40%	38%	52%	46%	48%	43%
Hoarse voice	14%	15%	14%	15%	13%	13%	10%	13%	16%	16%	14%	15%
Confusion	3%	3%	3%	3%	5%	6%	1%	2%	4%	3%	3%	3%
Diarrhoea	8%	11%	8%	11%	7%	8%	10%	15%	7%	9%	8%	11%
Fever	23%	25%	23%	25%	37%	39%	31%	30%	19%	22%	23%	25%
Persistent Cough	19%	24%	19%	24%	24%	27%	21%	25%	18%	23%	19%	24%
Anosmia	12%	10%	12%	10%	44%	35%	8%	8%	14%	11%	12%	10%
Dizzyness	14%	13%	14%	13%	24%	20%	5%	9%	19%	15%	14%	13%
Eye soreness	8%	8%	8%	8%	20%	19%	5%	7%	10%	8%	8%	8%
Red welts	3%	2%	3%	2%	3%	3%	4%	3%	2%	1%	3%	2%
Blisters	2%	1%	2%	1%	2%	1%	2%	1%	2%	2%	2%	1%
Nausea	17%	19%	17%	19%	17%	16%	14%	17%	19%	20%	17%	19%

**Illness duration**	5 [2;10]	4 [2;9]	8 [4;13]	7 [3;11]	7 [3;12]	6 [3;10]	3 [2;6]	3 [2;6]	3 [1;5]	3 [1;5]	3 [2;6]	3 [2;6]

Supplementary Table 7. Number of subjects reporting each symptom over the course of illness in younger (5-11 years, n=588), older (12-17 years, n=1,146) and overall (n=1,734) children testing negative for SARS-CoV-2. Data refers to children with symptom onset between 1 September 2020 and 24 January 2021.

	**Cohort testing negative for SARS-CoV-2**		
	**Younger children (aged 5-11 years, n=588)**	**Older children (aged 12-17 years, n=1,146)**	**Overall cohort (n=1,734)**
Headache	228	559	787 (45.4%)
Fatigue	158	426	584 (33.7%)
Sore Throat	274	695	969 (55.9%)
Anosmia	45	142	187 (10.8%)
Fever	179	234	413 (23.8%)
Abdominal Pain	145	176	321 (18.5%)
Dizziness	43	193	236 (13.6%)
Persistent Cough	137	236	373 (21.5%)
Loss of Appetite	67	148	215 (12.4%)
Eye Soreness	37	101	138 (8.0%)
Myalgias	34	114	148 (8.5%)
Nausea	90	219	309 (17.8%)
Hoarse voice	68	185	253 (14.6%)
Chest Pain	21	104	125 (7.2%)
Dyspnoea	21	70	91 (5.2%)
Diarrhoea	71	88	159 (9.2%)
Confusion	8	40	48 (2.8%)
Red Welts	19	20	39 (2.2%)
Blisters	8	20	28 (1.6%)

Supplementary Table 8. Official data of SARS-CoV-2 positive cases in children and young adults in England, Scotland, Wales and Northern Ireland.

	**Number of positive tests**	**Age [years]**	**Period**	Number of positive tests in defined age groups	Age [years]
**England**[1]	390,866	5-19	1 September 2020 to 24 January 2021	69,641	5-9
				321,225	10-19
**Scotland**[2]	30,466	0-19	Start of COVID-19 pandemic to 30 March 2021	15,869	0-14
				14,597	15-19
**Wales**[3]	~28,300	children and young people	Start of COVID-19 pandemic to 30 March 2021	-	-
**Northern Ireland**[4]	13,268	0-19	Start of COVID-19 pandemic to 30 March 2021	-	-

Supplementary References:

1. Public Health England. Weekly national Influenza and COVID-19 surveillance report - Week 11 report (up to week 10 data) 18 March 2021. https://assets.publishing.service.gov.uk/government/uploads/system/uploads/attachment_data/file/971212/Weekly_Flu_and_COVID-19_report_w11_v2.pdf (accessed April 27, 2021).
2. Public Health Scotland. Total Cases by Age and Sex. 2021. https://www.opendata.nhs.scot/fa_IR/dataset/covid-19-in-scotland/resource/19646dce-d830-4ee0-a0a9-fcec79b5ac71
3. Public Health Wales TP. Rapid COVID-19 surveillance. https://public.tableau.com/profile/public.health.wales.health.protection#!/vizhome/RapidCOVID-19virology-Public/Headlinesummary. 2021; published online April 16.
4. Ulster University. Northern Ireland COVID-19 Tracker - Cumulative Number of Individuals Tested Positive by Age. 2021. https://www.ulster.ac.uk/coronavirus/research/impact/ni-covid-19-tracker.

Supplementary Figure 1. Proportions of children with a positive test for SARS-CoV-2 with symptoms from questions added to the app, from 4 November 2020. Data refers to children with symptom onset between 1 September 2020 and 24 January 2021.

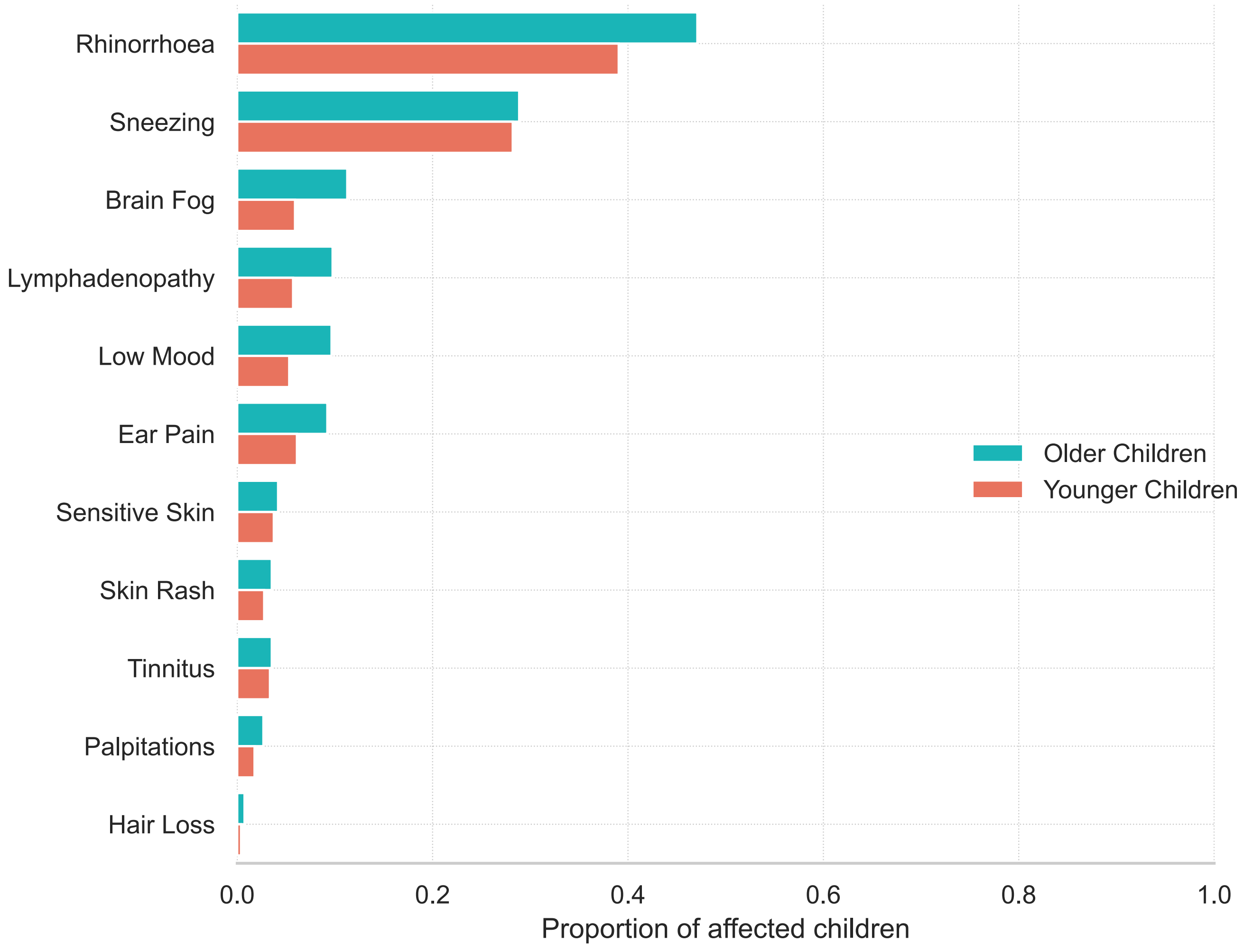

Supplementary Figure 2. Individual symptom profile and prevalence in younger children (YC, left panel) and older children (OC, right panel) testing positive for SARS-CoV-2, comparing 37 (16 younger, 21 older) children presenting to hospital (darker bars) with children managed in the community (lighter bars). Data refers to children with symptom onset between 1 September 2020 and 24 January 2021.

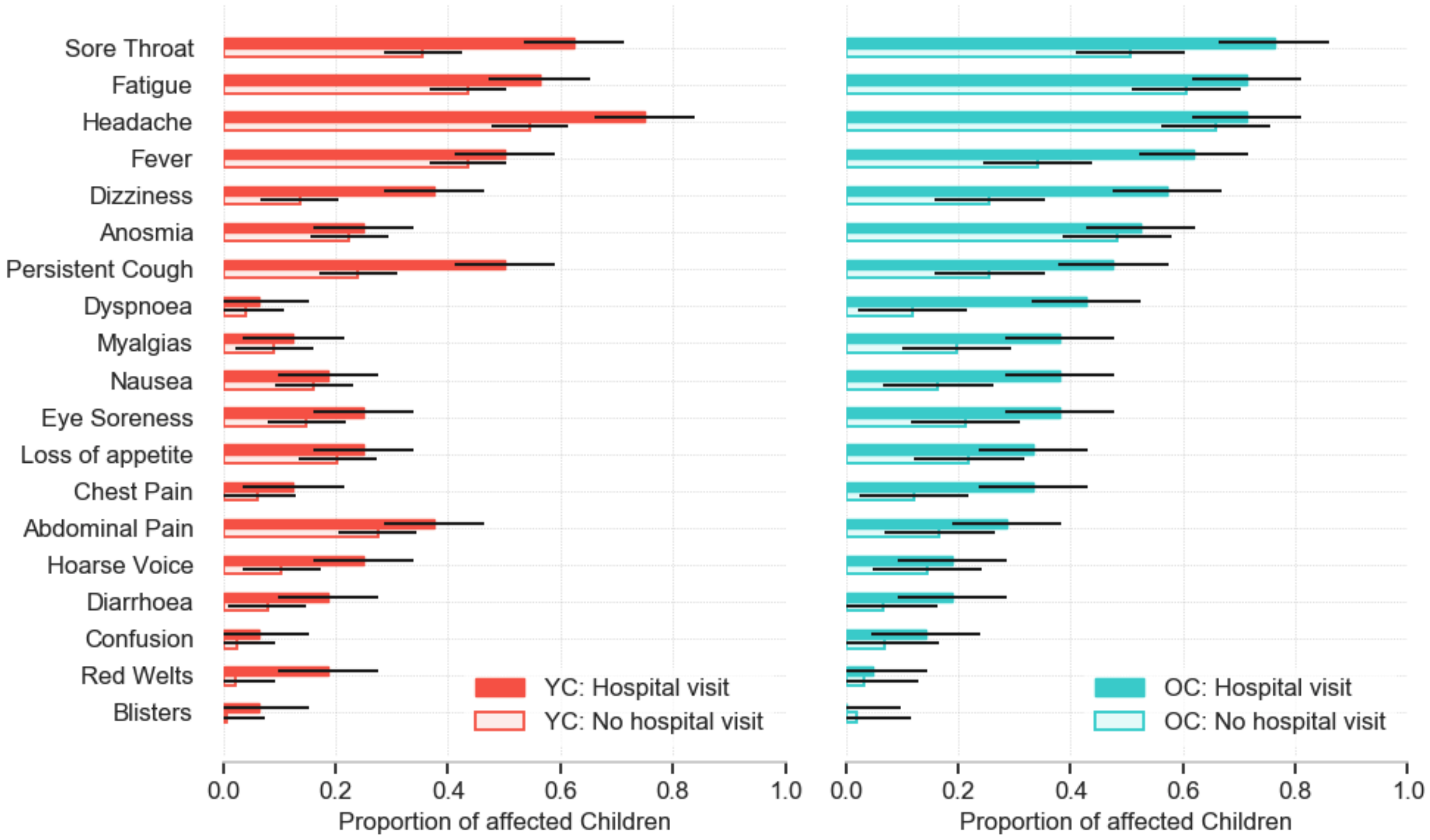

Supplementary Figure 3. Median symptom duration [IQR] in the control cohort (matched for age, gender, and week of testing) in younger (5-11 years) and older (12-17 years) children negative for SARS-CoV-2 (n=1734). Data refers to children with symptom onset between 1 September 2020 and 24 January 2021.

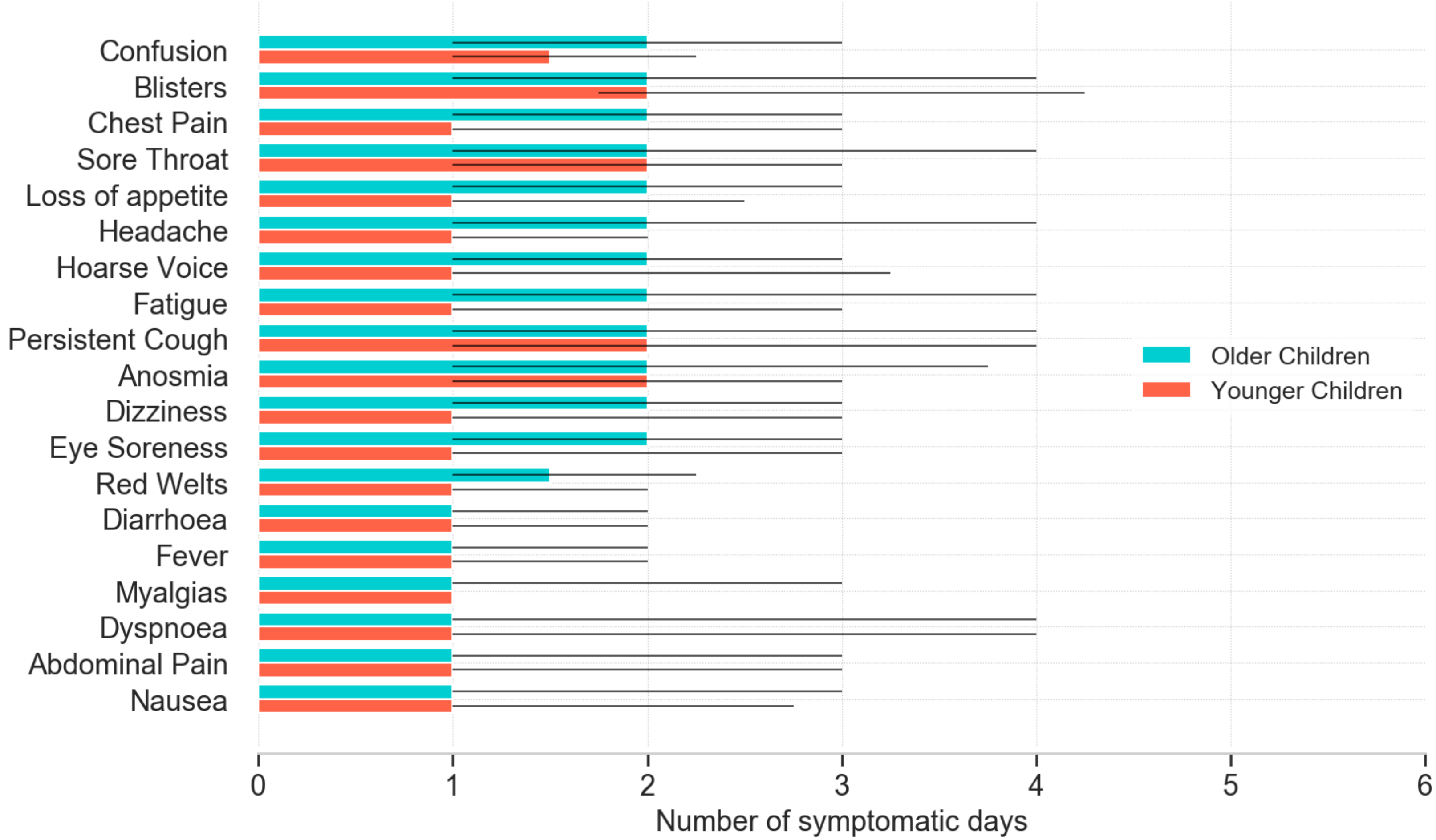

Supplementary Figure 4. Heat map showing symptom duration in children (aged 5-17 years) with a negative SARS-CoV-2 test, in whom **at least one symptom persisted for ≥28 days (n =15 children).** Data refers to children with symptom onset between 1 September 2020 and 29 December 2021.

Legend:

X-axis, duration in days.

Y axis, symptoms.

Legend: BT, blisters; RW, red welts; ES, eye soreness; DZ, dizziness and light-headedness; AN, anosmia; PC, persistent cough; FV, fever; DI, diarrhoea; CO, confusion; HV, hoarse voice; HA, headache; UMP, myalgias [unusual muscle pains]; LA, loss of appetite; SOB, dyspnoea [shortness of breath]; ST, sore throat; CP, chest pain; AP, abdominal pain; FA, fatigue. Colour bar provides percentage comparison.

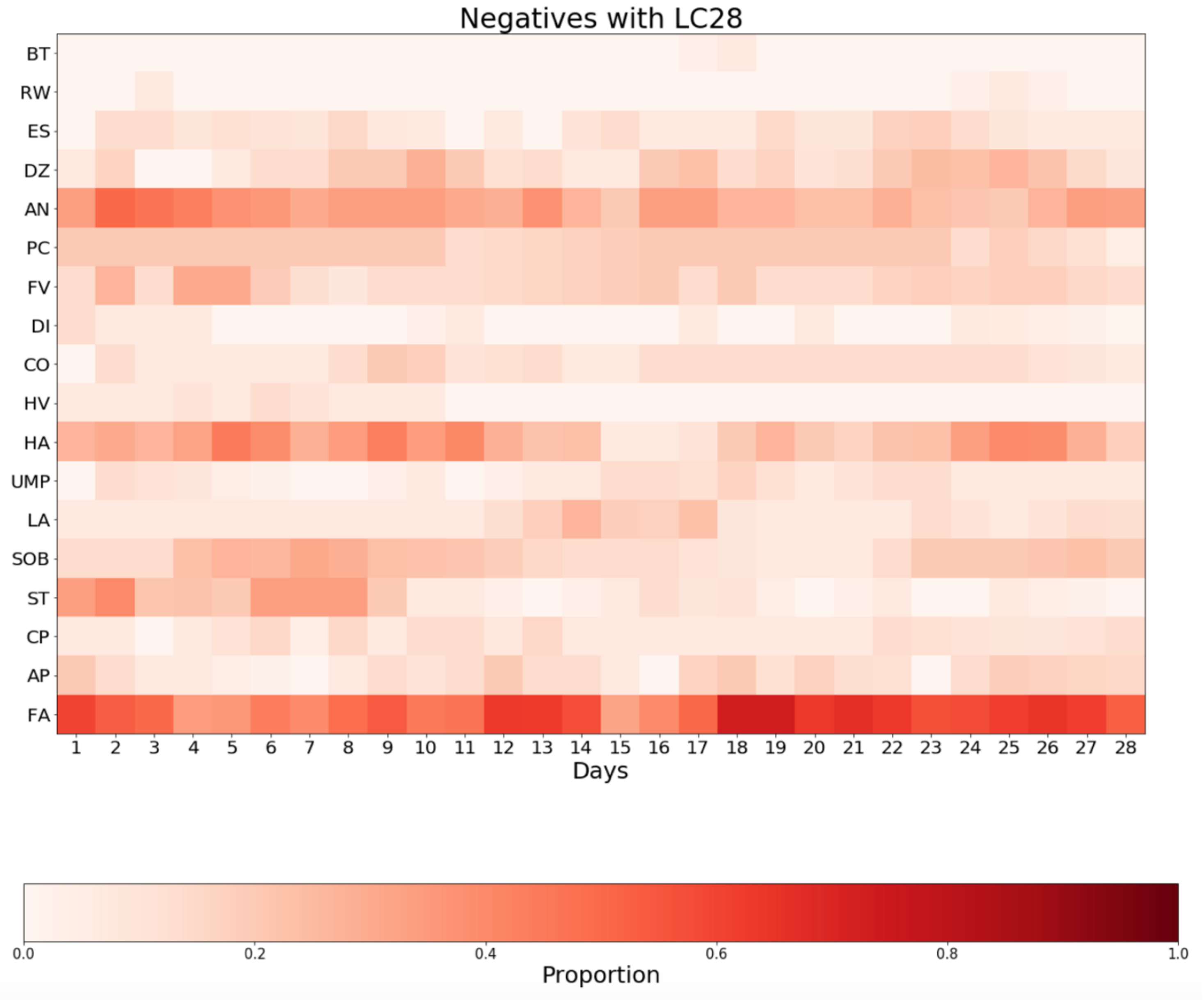
Negatives with LC28
BT
RW
ES
DZ
AN
PC
FV
DI
CO
HV
HA
UMP
LA
SOB
ST
CP
AP
FA
1 2 3 4 5 6 7 8 9 10 11 12 13 14 15 16 17 18 19 20 21 22 23 24 25 26 27 28
Days
0.0 0.2 0.4 0.6 0.8 1.0
Proportion

Supplementary Figure 5. Symptom profile (at day 28 or beyond) in younger children (YC, left panel) and older children (OC, right panel) with illness duration ≥28 days. Each panel compares children positive (darker bars) and negative (lighter bars) for SARS-CoV-2. Data refers to children with symptom onset between 1 September 2020 and 29 December 2021.

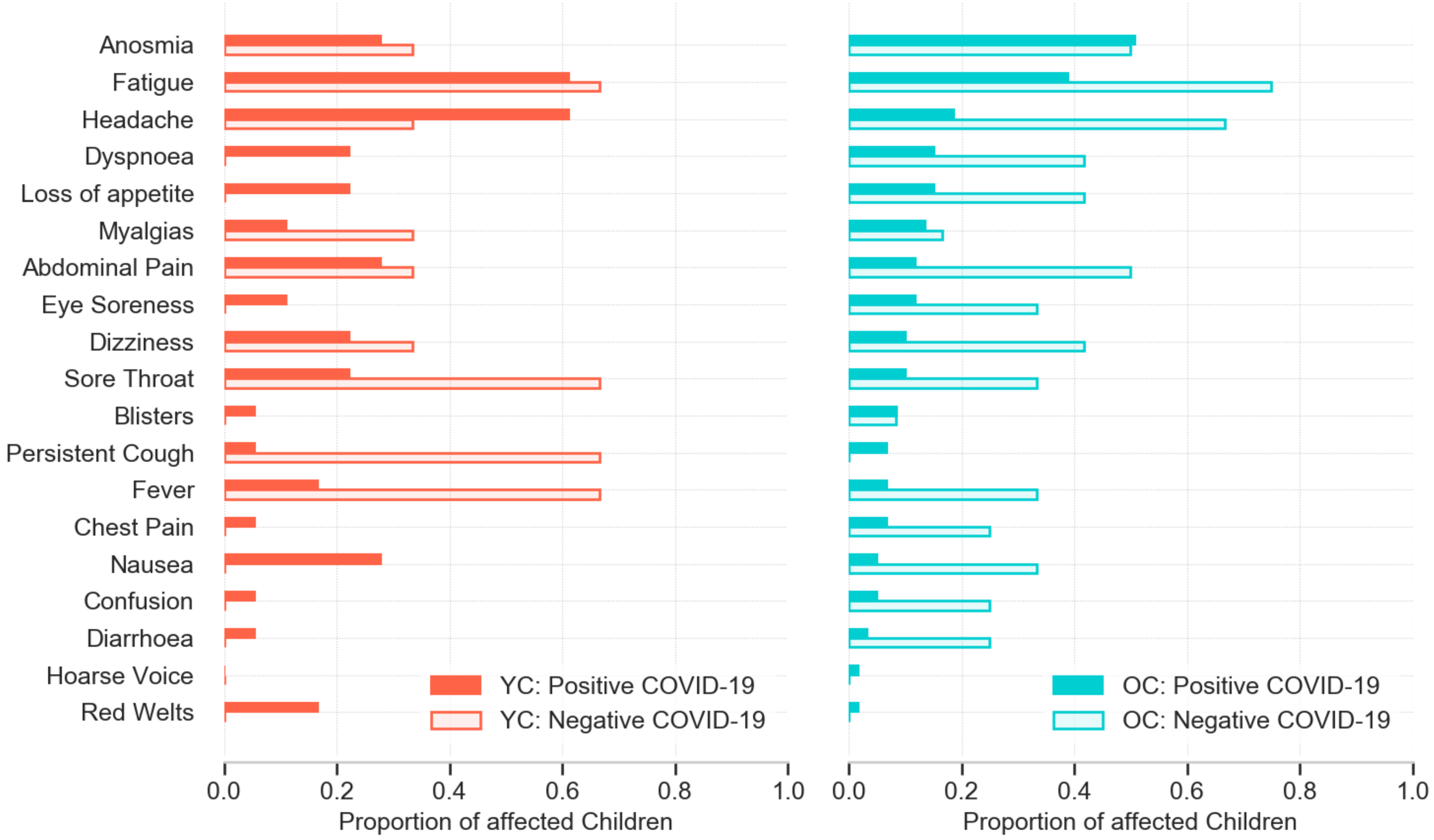

Supplementary Figure 6. Symptom prevalence over the entire duration of illness in younger children (YC, left panel) and older children (OC, right panel) with illness duration >28 days, comparing children who tested positive (darker bars) or negative (lighter bars) for SARS-CoV-2. Data refers to children with symptom onset between 1 September 2020 and 29 December 2021.

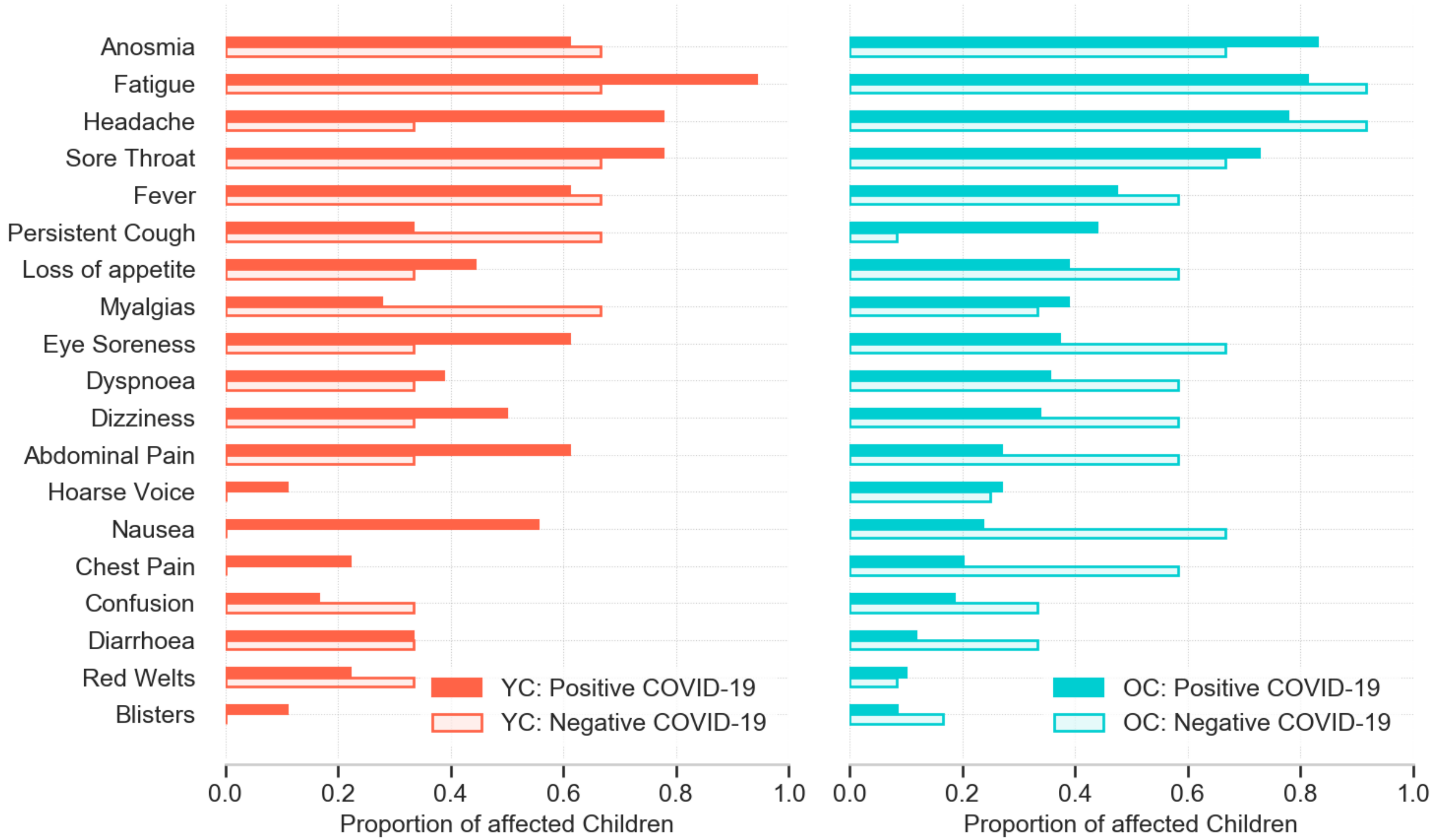

Supplement: Supplementary appendix [file mmc1.pdf]
